# A Comparative Systematic Review of the Optimal CD4 Cell Count Threshold for HIV Treatment Initiation

**DOI:** 10.1155/2014/625670

**Published:** 2014-03-20

**Authors:** Babatunde Olubajo, Kathryn Mitchell-Fearon, Oluseye Ogunmoroti

**Affiliations:** ^1^Eastern Health Research and Analysis, Inc., 1 Press Place, Athens, GA 30601, USA; ^2^Department of Community Health and Psychiatry, The University of the West Indies, Mona, Kingston 7, Jamaica; ^3^Atlanta English Institute, 4000 Dekalb Technology Parkway, Atlanta, GA 30340, USA

## Abstract

HIV infection is no longer characterized by high morbidity, rapid progression to AIDS, and death as when the infection was first identified. While anti-retroviral drugs have improved the outcome of AIDS patients, clinical research on the appropriate time to initiate therapy continues to evolve. Optimal therapy initiation would maximize the benefits of these drugs, while minimizing side effects and drug resistance. Recent 2013 WHO guidelines changed HIV therapy initiation from 350 cells/**μ**L to 500 cells/**μ**L. This systematic review provides an evidence-based comparison of starting treatment at >500 cells/**μ**L with starting treatment at the range between 350 cells/**μ**L and 500 cells/**μ**L. An 11% increase in risk was detected from initiation therapy at the 350–500 cells/**μ**L range (0.37 [0.26, 0.53]), when compared with starting treatment before 500 cells/**μ**L (0.33 [0.22, 0.48]). Most individual study comparisons showed a benefit for starting treatment at 500 cells/**μ**L in comparison with starting at the 350–500 cells/**μ**L range with risks ranging from 19% to 300%, though a number of comparisons were not statistically significant. Overall, the study provides evidence based support for initiating anti retroviral therapy at cell counts >500 cells/**μ**L wherever possible to prevent AIDS mortality and morbidity.

## 1. Introduction

Decades of institutional HIV research led to effective therapies that allow for the management of HIV infection like a chronic disease. Unlike the earliest HIV cases, infected individuals live longer due to antiretroviral therapy (ART) drug combinations.

Azidothymidine (AZT), the first approved antiretroviral, represented a breakthrough in the treatment of HIV/AIDS [1] in the 1990s but was later replaced with potent reverse transcriptase inhibitors which include nucleoside and nucleotide drugs. The development of these replication inhibitor drugs combined with protease, integrase, and cell entry inhibitors now form the HIV treatment regimen that has reduced the incidence of HIV progression to AIDS in many patients.

Currently, the standard and most effective delivery of ART is in the form of highly active antiretroviral therapy (HAART), consisting of a three to four drug combination from the six classes of ARTs [2]. The main classes of ART drugs—nucleoside, nucleotide, and nonnucleoside reverse transcriptase inhibitors—as well as integrase strand transfer inhibitors disrupt HIV genome replication machinery. The remaining ART drug classes disrupt viral entry into host cells (fusion inhibitors, CCR5 antagonists) or disrupt the function of other proteins integral to viral development (protease inhibitors). Combinatorial HIV drug treatment strategies maintain low viral levels in patients [3, 4] and thus reduce HIV transmission [5, 6].

The World Health Organization (WHO) recommends a first-line ART should consist of two nucleoside reverse transcriptase inhibitors (NRTIs) plus a nonnucleoside reverse transcriptase inhibitor (NNRTI) [7]. The United States Department of Health and Human Services (USDHHS) recommends a similar first-line regimen with two NRTIs and either a NNRTI, a protease inhibitor boosted with ritonavir, an integrase inhibitor, or a CCR5 antagonist [2]. The WHO and the USDHHS give these regimens a “strong” recommendation based on analysis of HIV treatment data, clinical research literature, and expert endorsement [2, 7].

CD4+ T lymphocyte (CD4) cell counts are the primary laboratory markers used to track the progression of HIV to AIDS; however, clinicians still debate the appropriate CD4 threshold at which to initiate HIV therapy. The 2013 WHO and the USDHHS guidelines recommend HIV therapy at CD4 cell counts less than 500 cells/*μ*L, a recent departure from the prior guidelines that gave 350 cells/*μ*L the strongest recommendation. While the therapy should be initiated based on individual patient characteristics, societal factors such as resource availability of health staff and a continuous supply of drugs must also be considered before initiating therapy [8]. The debate of when to initiate therapy is also fueled by the lack of evidence from HIV “treatment initiation” randomized clinical trials (due to ethical implications). The scientific literature does, however, include observational treatment initiation studies of varying quality that can bring treatment guidelines closer to the best treatment strategy.

To further clarify initiation of HIV therapy with ART drugs and conduct an evidence based analysis of HIV treatment initiation we conducted a systematic review of observational HIV treatment initiation studies. We hypothesized that initiation at >500 cells/*μ*L will lead to a reduction in risk of patient mortality or a progression to AIDS when compared with initiation at the 350–500 cells/*μ*L range. To our knowledge, this is the first comparison in the scientific literature of HIV therapy initiation between these two subgroups through an observational study systematic review.

## 2. Methods

### 2.1. Inclusion/Exclusion Criteria

Studies included in this systematic review were all cohort studies which assessed CD4 count threshold, defined progression to AIDS, AIDS defining events (ADE), or mortality following therapy initiation with ART or HAART. The studies included adolescents and adults and excluded pregnant women. The effect measure chosen to compare outcomes was hazard ratios, and the particular ART or HAART regimen was not relevant. All included studies were published in English and between the years 2000 and 2013. Subanalysis of CD4 cell count ranges and the referent group chosen for comparison were also cause for elimination. Several large studies had overlapping subcohorts and some studies compared early to deferred treatment within the same subgroup which was not the focus of this review.

### 2.2. Literature Search Strategy

A systematic and comprehensive literature search was undertaken by developing an effective search strategy. PubMed, MEDLINE, and EBSCO databases were searched, using the text words CD4, treatment, initiation, threshold, HAART, HIV, and Anti-retro* with limited results. PubMed revealed the best results, using the MeSh terms “Anti retroviral agents/therapeutic use,” “Anti retroviral therapy, Highly active,” “CD4 lymphocyte count,” “HIV infections/mortality,” and “Treatment outcome.” After using Boolean search terms “Initiating AND HAART,” “HAART AND CD4 AND Treatment outcome,” we found that the terms with “HAART,” “CD4,” and the threshold number “500” produced the best results. Two investigators independently reviewed abstracts/papers produced by the aforementioned search strategy and systematically included/excluded studies from the review.

### 2.3. Quality Assessment and Data Analysis

Most systematic review instruments for assessing study quality were developed for nonrandomized studies and were thus unsuitable for this project. Hence, the Newcastle-Ottawa Scale (NOS) [16] was used to assess the quality of the observational studies used in this analysis. The NOS was developed by collaboration between the University of Newcastle, Australia, and Ottawa, Canada, and it assesses the study quality of nonrandomized studies. Questions used in this tool assessed various “quality” determining aspects of each study. The major assessment areas were (1) the selection of study cohorts, (2) comparability of study groups, and (3) methods of determining study outcomes. For studies where appropriate, the Cochrane Collaboration's Review Manager 5.2 was used to pool study effect measures using the inverse variance method in a fixed effects model and to calculate the *I* ^2^ statistic of study heterogeneity.

## 3. Results and Discussion

**Figure 1:**
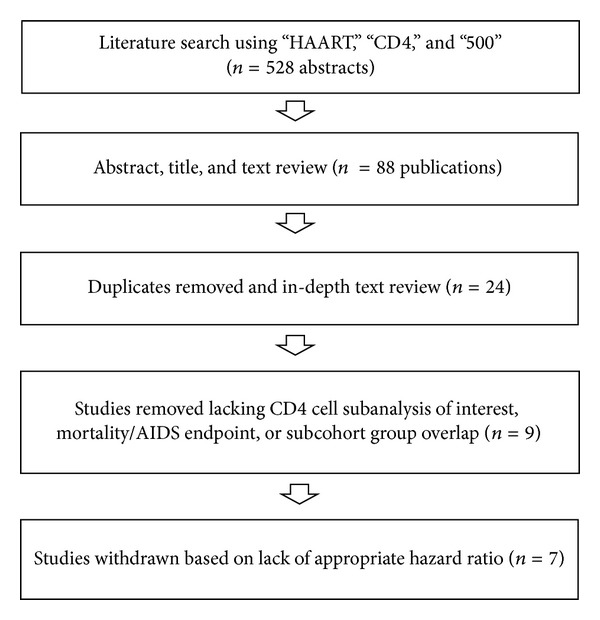
Flow chart of literature search strategy.

The literature search produced 528 abstracts from which the final studies were selected. The PubMed search using “HAART,” “CD4,” and “500” produced abstracts that were reviewed by two authors with some publication text review for further confirmation. Eighty-eight publications were reviewed in a conference by two authors removing duplicates and articles that did not fit the review topic, leaving twenty-four publications. Fifteen studies were removed from the 24 because they lacked either the subanalysis of interest (>500 cells/*μ*L, 350–500 cells/*μ*L) or a mortality/AIDS endpoint. Further, some of the 15 studies eliminated at this point compared initiation to deferment of treatment within a particular subgroup and some large studies with multiple subcohorts had overlapping subcohorts. Of the remaining nine studies, two were eliminated for not having appropriate effect measures (i.e., HR) for comparison. The flow chart in Figure 1 shows the search strategy described and Table 1 shows the final 7 papers used in the review.

As mentioned in the methods section, the Newcastle-Ottawa scale assessed study quality by giving a “star” for successfully meeting criteria of the scale. We assessed “5 stars” as a reasonable cutoff and the seven studies received 5 stars or greater on the Newcastle-Ottawa scale.

Data from each of the seven studies was elicited into two distinct categories: (1) patients who initiated ART/HAART at CD4 counts at or above 500 cells/*μ*L and (2) patients who initiated ART/HAART at CD4 counts between 350 and 500 cells/*μ*L.

One issue arose throughout the review—identifying articles that presented results of treatment initiation stratified by our particular subgroups of interest. For example, for the Causal 2011 [15] study we compared 500 cells/*μ*L to 450 cells/*μ*L (the closest subgroup).

Table 2 summarizes the data from all the studies, showing the hazard ratios (HR) and confidence intervals (CIs) for both categories of each study (i.e., >500 cells/*μ*L and 350–500 cells/*μ*L). The hazard ratios measured the risk associated with progression to AIDS or mortality when initiating therapy at the predefined CD4 count. It is important to note that the first three studies in Table 2 used cases with a CD4 count less than 200 cells/*μ*L as the referent group, while the four remainder studies used cases with 500 cells/*μ*L as the referent group. The 500 cells/*μ*L referent group studies [12–15] exhibited an increased risk for initiation at 350–500 cells/*μ*L, but only 2 of the studies had statistically significant comparisons [12, 15]. We could not pool the effect measures of these 2 studies because they did not have a similar breakdown of CD4 subgroup ranges.

In the 200 cells/*μ*L referent group studies [9–11], the 350 to 500 cells/*μ*L group and the >500 cells/*μ*L group both showed a significant reduction in risk for mortality/AIDS; this was consistent across the three studies by Garcia, Griensven, and Lelyveld (Table 2). These studies indicate that 500 cells/*μ*L and 350–500 cells/*μ*L are better thresholds for initiating therapy than at 200 cells/*μ*L but with caution that there is no statistically significant difference between starting at 500 or at 350–500 as the 95% confidence intervals overlap.

Based on the Kaplan study [12], when the 350–500 cells/*μ*L group is compared to the 500 cells/*μ*L referent group, there was a significantly higher risk for mortality/AIDS (Table 2); in fact the risk of a poor outcome was 3.1 times larger in those initiating therapy at 350–500 cells/*μ*L compared to initiating therapy at greater than 500 cells/*μ*L. This supports the findings of García et al. [9], Van Griensven and Thai [10], and Van Lelyveld et al. [11] that initiating therapy at a higher CD4 count (i.e., >500 cells/*μ*L) will reduce the risk for a poor outcome. The Kaplan study seems to be more sensitive in picking up differences between the 500 cells/*μ*L and the 350–500 cells/*μ*L categories. The studies by Kawado et al. [13] and Lifson et al. [14] did not show a statistically significant difference between initiating treatment at 350–500 cells/*μ*L or at >500 cells/*μ*L.

**Figure 2:**
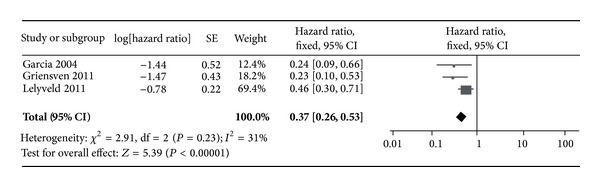
Forrest plot of >500 cells/*μ*L group hazard ratios.

**Figure 3:**
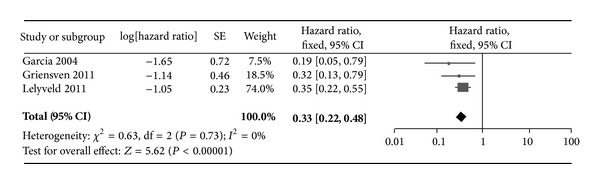
Forrest plot of 350–500 cells/*μ*L group hazard ratios.

The studies with groups of patients categorized and compared by thresholds >500 cells/*μ*L and 350–500 cells/*μ*L were separated and the groups were pooled by group to produce a composite effect measure (Figures 2 and 3). A total of 6 subgroups were pooled and compared according to CD4 threshold. As shown in Figures 2 and 3, the pooled hazard ratio was 0.33 [0.22, 0.48] for the >500 cells/*μ*L group; this is a larger (though nonsignificant) risk reduction than that found in those initiating therapy at the 350 to 500 cells/*μ*L group (0.37 [0.26, 0.53]).

*I* ^2^ statistic values of 0% for the >500 cells/*μ*L group and 31% for the 350 to 500 cells/*μ*L group indicate sufficient homogeneity to proceed with this analysis; publication bias was not assessed for the 3 studies.

## 4. Conclusions

Changes in HIV therapy initiation guidelines affect clinicians, patients, and policymakers who continue to search for the most efficient and effective treatment strategies. Prior to 2013, initiation was recommended at 350 cells/*μ*L [17] and developing countries—where governments and international agencies play a greater role in HIV management due to low per capita income on the part of patients—are still adjusting to these changes. The WHO and USDHHS in 2013 recommended starting therapy at >500 cells/*μ*L based on the scientific body of science for HIV clinical research. Some support for this change in recommendation may be provided through this meta-analysis of studies that compares the former recommendation (initiation at <350 cells/*μ*L) to the new recommendation (>500 cells/*μ*L) but only when <200 cells/*μ*L are used as a referent group.

In resource limited countries, where increasing the threshold for initiating treatment to CD4 counts of 350 cells/*μ*L is financially very difficult, the WHO guidelines may be met with resistance and consequently may not be adopted and adhered to. This review gives insight to the risk associated with maintaining a low CD4 threshold as compared to the elevated threshold recommended by WHO and the USDHHS. Results from this review indicated a greater risk in those initiating therapy at 350–500 cells/*μ*L compared to those initiating therapy at CD4 >500 cells/*μ*L, but only with the studies that were appropriate for combining the effect. Of the studies with <200 cells/*μ*L as a referent group, there was a pooled 11% elevated risk for the 350–500 cells/*μ*L cells; only one of the three studies exhibited a decreased risk for initiating therapy at 350–500 cells/*μ*L as opposed to initiating at >500 cells/*μ*L.

This review may only be used to provide supportive and not definitive evidence because the difference between study groups was not significant for two of the studies in the 500 referent group and one study did not have a similar subgroup to compare. More studies that look at >500 cells/*μ*L or 350–500 cells/*μ*L as referent groups are needed for firm clarification of the benefits or risk. Overall, this review suggests that, whenever possible, therapy should be started when CD4 counts are at or above 500 cells/*μ*L rather than waiting for the CD4 to fall to lower counts or the old recommended guidelines (350 cells/*μ*L) in order to prevent mortality and morbidity due to AIDS progression.

## Figures and Tables

**Table 1:** Descriptive characteristics of final studies used for systematic review.

Author	Study year	Total pop. *N*	Followup	>500 *N*	350–499 *N*
García et al. [9]	2004	861	8 months	90	137
Van Griensven and Thai [10]	2011	2840	6 months	N/A	N/A
Van Lelyveld et al. [11]	2012	3068	24 months	811	1413
Kaplan et al. [12]	2003	2729	21 months	499	483
Kawado et al. [13]	2006	605	60 months	114	134
Lifson et al. [14]	2012	1590	104.4 months	279	433
Cain et al. [15]	2011	20971	N/A	N/A	N/A

N/A stands for not available.

**Table 2:** Effect measure and referent group of studies reviewed.

Author	Reference (cells/*μ*L)	>500 cells/*μ*L HR	<500 HR cells/*μ*L HR (350–500 cells/*μ*L, *450 cells/*μ*L)
García et al. [9]	<200	0.192 (0.047–0.79)	0.238 (0.086–0.662)
Van Griensven and Thai [10]	<200	0.32 (0.13–0.79)	0.23 (0.10–0.53)
Van Lelyveld et al. [11]	<200	0.35 (0.22–0.55)	0.46 (0.30–0.70)
Kaplan et al. [12]	>500	1	3.1 (1.4–6.6)
Kawado et al. [13]	≥500	1	1.36 (0.45–4.16)
Lifson et al. [14]	≥500	1	1.29 (0.71–2.35)
Cain et al. [15]	>500	1	*1.14 (1.07–1.22)
